# Supplementary figures and images for: Spatiotemporal distribution and sociodemographic and socioeconomic factors associated with primary and secondary syphilis in Guangdong, China, 2005–2017
Source: PLoS Negl Trop Dis. 2021 Aug 12;15(8):e0009621. doi: 10.1371/journal.pntd.0009621 (PMC8407558; doi:10.1371/journal.pntd.0009621)

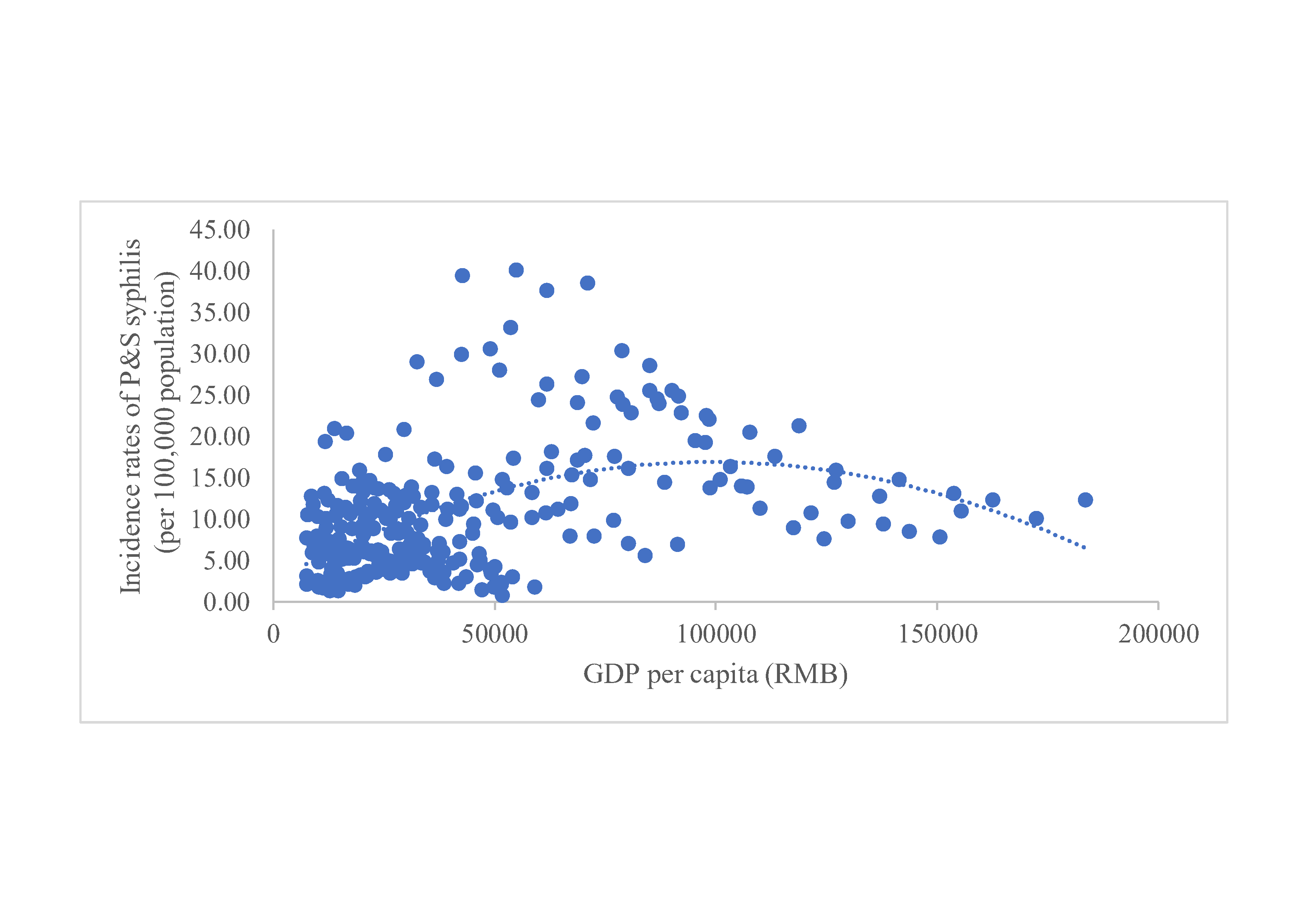

Supplement: S1 Fig — (TIFF) [file pntd.0009621.s001.tiff]

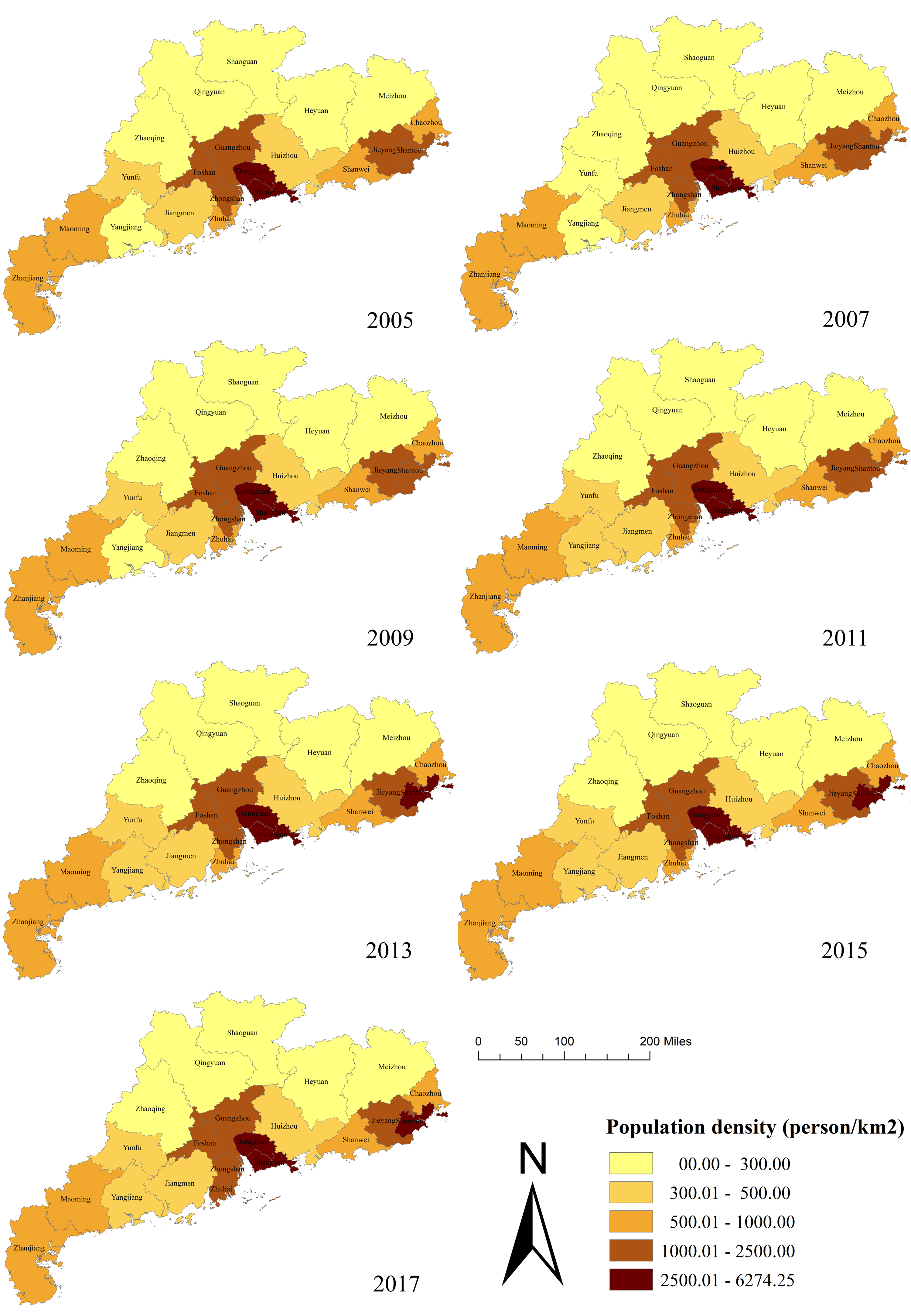

Supplement: S2 Fig — Base layers of the maps were downloaded from Resource and Environment Science and Data Center (http://www.resdc.cn/data.aspx?DATAID=201). (TIF) [file pntd.0009621.s002.tif]

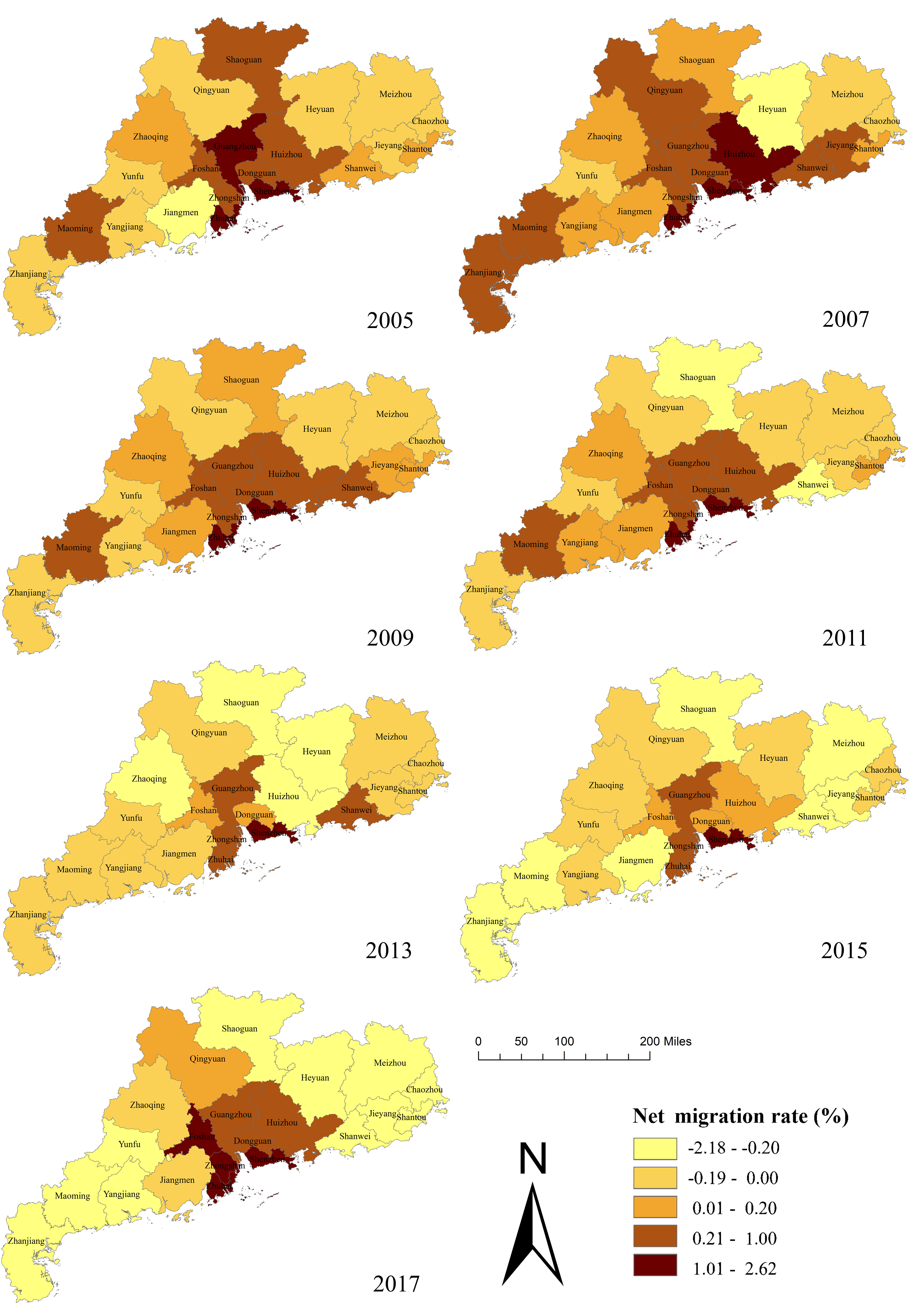

Supplement: S3 Fig — Base layers of the maps were downloaded from Resource and Environment Science and Data Center (http://www.resdc.cn/data.aspx?DATAID=201). (TIF) [file pntd.0009621.s003.tif]

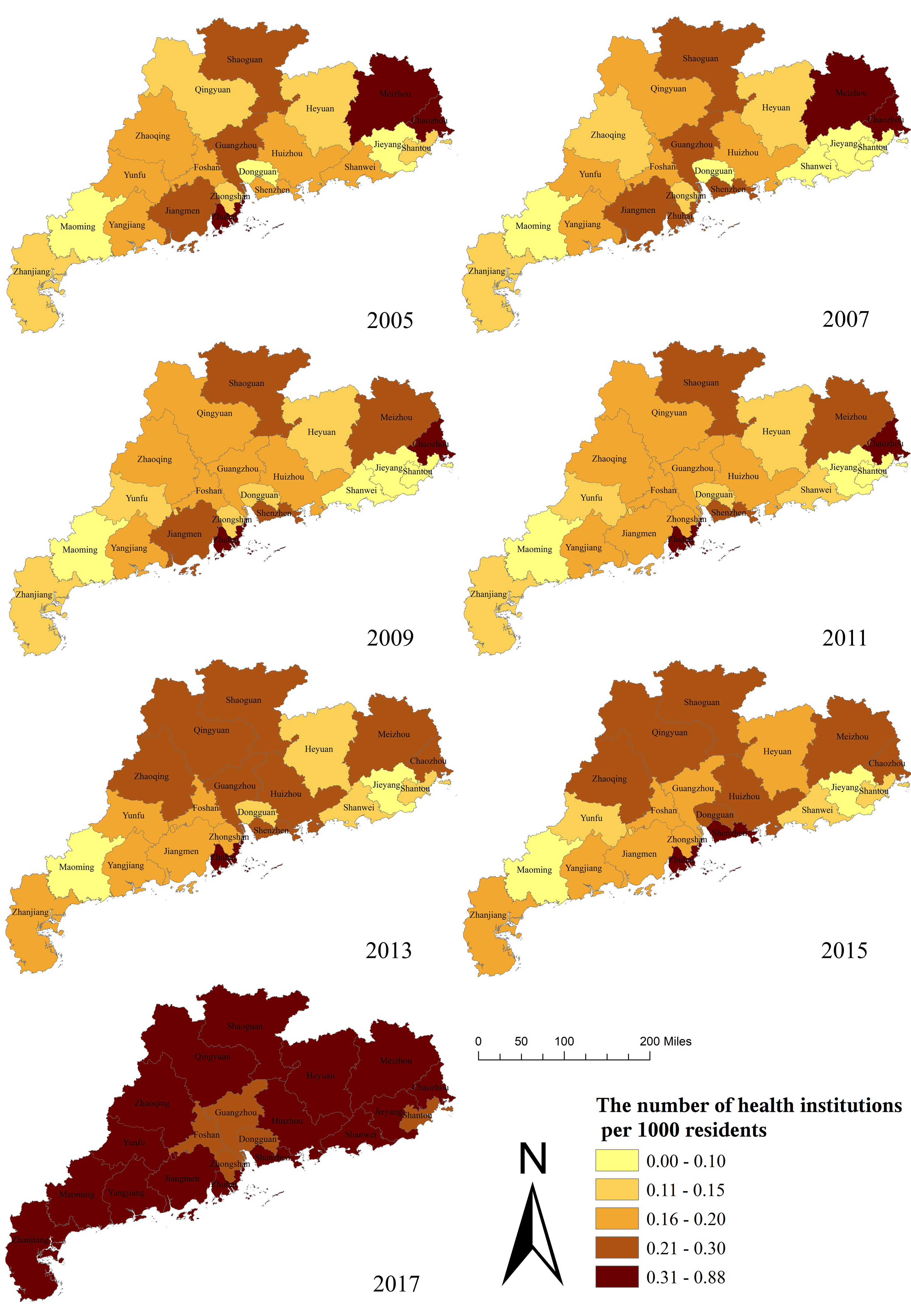

Supplement: S4 Fig — Base layers of the maps were downloaded from Resource and Environment Science and Data Center (http://www.resdc.cn/data.aspx?DATAID=201). (TIF) [file pntd.0009621.s004.tif]

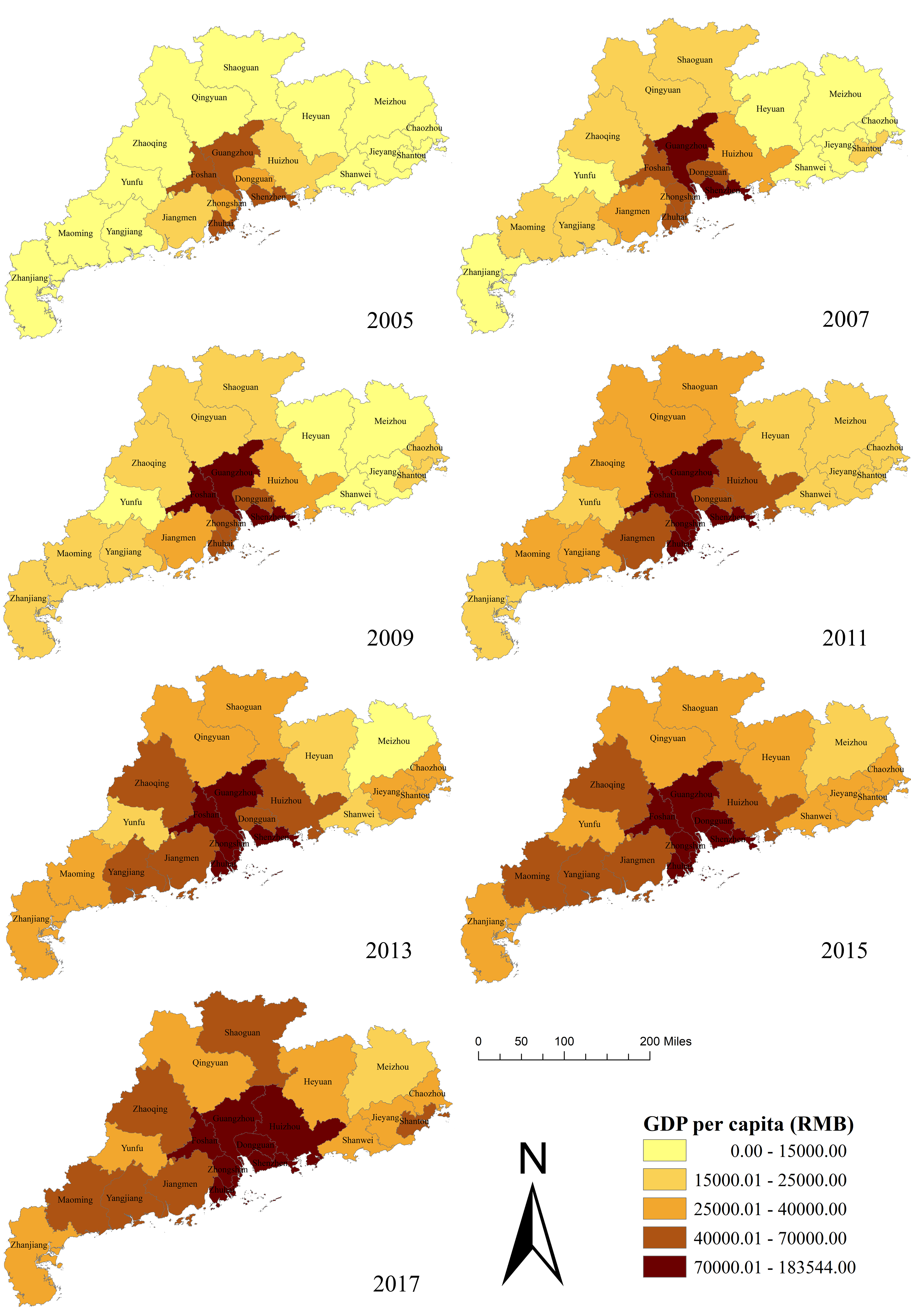

Supplement: S5 Fig — Base layers of the maps were downloaded from Resource and Environment Science and Data Center (http://www.resdc.cn/data.aspx?DATAID=201). (TIF) [file pntd.0009621.s005.tif]
