# Supplementary material for: Spatiotemporal distribution and sociodemographic and socioeconomic factors associated with primary and secondary syphilis in Guangdong, China, 2005–2017
Source: PLoS Negl Trop Dis. 2021 Aug 12;15(8):e0009621. doi: 10.1371/journal.pntd.0009621 (PMC8407558; doi:10.1371/journal.pntd.0009621)
Supplement: Attachment — Submitted filename: Review_Apr20.docx [file pntd.0009621.s006.docx]

General comments:

This is a relatively simple and straightforward study that provides needed insight regarding the sociodemographic and socioeconomic factors surrounding P&S Syphilis transmission.

There is quite a bit of tense-switching throughout the manuscript (past, present, and future tenses were all noted). Please check thoroughly

Specific comments:

Line 79: “Syphilis is a kind of bacterial infectious disease caused by” – change to “Syphilis is a bacterial infectious disease caused by”

Line 79: Italize Treponema pallidum,

Line 86: strike “entire”

Line 87: move (P&S) to first sentence.

Line 99: Find alternative words for “According to”, maybe “Regarding”

Line 99-101: Reconsider rewording this sentence: “Regarding socioeconomic factors, studies present conflicting results concerning incidence.”

Line 108: Change phrase to: “however, research is lacking to support this claim.”

Line 117: change “based on” to “ “while”

Line 121: Can you provide a map of the region both broad and fine scale (inset) to help the reader with the area?

Line 124: Start the sentence with, “In 2017, there was great…” and do not switch between tenses

Line 128: Check journal standards, but values less than 10 are spelled out, i.e., ‘nine’

Line 134: Does this surveillance system have an official name?

General: How often are cases reported? And what is the spatial resolution of those reports (to which level of governance are they aggregated?)

Line 137: “will be” or “was”? Check your use of tense throughout

Line 141: Do you mean ‘number of health institutions’?

Line 142: “person counts”? ‘number of people per square kilometer’ ?

Line 145: Add a comma after ‘structure’

Line 152: Replace ‘sunshine” with ‘daylight’

Lines 151-152: Provide the units in which these metrics were recorded. Were these data aggregated to the administrative units? If so, how?

Lines 203-205: “A peak period”? Is there a peak monthly period each year? Or were these data aggregated?

Line 207: Regarding the time series maps. It appears that the incidence rate hot spot moves from north to south regions, until the rate nearly dissolves. What is this pattern I’m seeing?

Line 208: The term ‘hot spot’ is used here, but it has a specific meaning in spatial analyses that were not conducted in this study. It would be best to find a different term.

Line 225: There is an unnecessary period after 1.17

Line 237: Change ‘autoregressive’ to ‘autoregression’

Line 254: Add ‘factors’ after sociodemographic

Spatial clusters identification: Does the algorithm determine grouping of years, or was this done by the authors?

Results: Avoid redundancy by either 1) putting your statistics in a table OR 2) adding them to the body of the text. Generally, you should not do both. Example Line 230

Results: I recommend that you create a map (or series of maps) that shows distribution of your variables (e.g., population density, net migration, health institutions, etc.). It would be very helpful to visualize these data spatially. It could just be an appendix or supplemental figure.

Results: Can you graphically present the U-shaped relationships you found in this study?
