# Supplementary material for: Spatiotemporal distribution and sociodemographic and socioeconomic factors associated with primary and secondary syphilis in Guangdong, China, 2005–2017
Source: PLoS Negl Trop Dis. 2021 Aug 12;15(8):e0009621. doi: 10.1371/journal.pntd.0009621 (PMC8407558; doi:10.1371/journal.pntd.0009621)
Supplement: Attachment — Submitted filename: Response to Reviewers.docx [file pntd.0009621.s007.docx]

Dear editor,

Thank you very much for your letter and all the comments and suggestions on our manuscript entitled “Spatiotemporal distribution and sociodemographic and socioeconomic factors associated with primary and secondary syphilis in Guangdong, China, 2005-2017” (PNTD-D-20-02077). We do appreciate the opportunity to revise and resubmit the manuscript. Several modifications were made (details as below) in response to the reviewers’ comments and hopefully the new version could be qualified to be published. Thank you again for the valuable comments!

**Reviewers’ comments:**

**Introduction**

**Point 1:**

Line 79: “Syphilis is a kind of bacterial infectious disease caused by” – change to “Syphilis is a bacterial infectious disease caused by”

Line 79: Italize Treponema pallidum,

Line 86: strike “entire”

Line 87: move (P&S) to first sentence.

Line 99: Find alternative words for “According to”, maybe “Regarding”

Line 99-101: Reconsider rewording this sentence: “Regarding socioeconomic factors, studies present conflicting results concerning incidence.”

Line 108: Change phrase to: “however, research is lacking to support this claim.”

Line 117: change “based on” to “while”

**Response 1**: Sorry for the above unclear presentation. We have revised this section accordingly (Line 70-105).

**Methods**

Reviewer #1: The objectives the study are clearly written, study design appropriate, sample size is ok for the study. The statistical analysis done supports the conclusion.

Reviewer #2: The objectives of this study were clearly presented and easily tested. The study design appears appropriate to the hypotheses tested.  This is a relatively simple and straightforward study that provides needed insight regarding the sociodemographic and socioeconomic factors surrounding P&S Syphilis transmission.

**Point1：**Line 121: Can you provide a map of the region both broad and fine scale (inset) to help the reader with the area?

**Response 1**: Thanks for the constructive comments. We have added a map of Guangdong Province (Fig.1, Line 119).

**Point2：**

Line 124: Start the sentence with, “In 2017, there was great…” and do not switch between tenses

**Response2**: We have added “From 2005-2017,” and have revised the tense (Line 111-112).

**Point3：**Line 128: Check journal standards, but values less than 10 are spelled out, i.e., ‘nine’

**Response3**: We have changed ‘9’ to ‘nine’ (Line 115).

**Point4：**Line 134: Does this surveillance system have an official name?
General: How often are cases reported? And what is the spatial resolution of those reports (to which level of governance are they aggregated?)

**Response4**: We thank the reviewer to point out this comment. The official name of this surveillance system is the web-based National Notifiable Infectious Disease Reporting Information System. Every medical institution at county level or above in China is obliged to report all diagnosed cases of P&S syphilis online within 24 hours after the patient's diagnosis. In this study, the number of P&S syphilis cases was counted at the municipal-level within Guangdong Province. We used the annual P&S notification rate (per 100,000 population) for all 21 cities in Guangdong Province as an outcome variable in spatiotemporal analyses.

We have added related information in Line 121-129.

**Point5：**Line 137: “will be” or “was”? Check your use of tense throughout

**Response5**: We have changed ‘will be’ to ‘was’ (Line 130) and checked the tenses of full text.

**Point6：**Line 141: Do you mean ‘number of health institutions’?
Line 142: “person counts”? ‘number of people per square kilometer’?
**Response6**: Sorry for the unclear presentation. We have changed the phrases to ‘the number of health institutions per 1000 residents’ (Line 137) and ‘number of people per square kilometer’ (Line 138).

**Point7：**Line 145: Add a comma after ‘structure’
Line 152: Replace ‘sunshine’ with ‘daylight’

**Response7**: We have added a comma after ‘structure’ (Line 142) and have changed ‘sunshine’ to ‘daylight’ (Line 148 and Line 257).

**Point8：**Lines 151-152: Provide the units in which these metrics were recorded. Were these data aggregated to the administrative units? If so, how?

**Response8**: We thank the reviewer and we have added the units. These data were aggregated to municipal units. The meteorological data of a city was calculated by taking the annual average value of all meteorological station data of each city. We have added related information in Line 150-152.

**Point9：**add to the details in the methods on the source of the primary and secondary syphilis data - are they publicly available? is there a web link? and if not, how would other researchers obtain these data?

**Response9**: The syphilis data analyzed in our study are not publicly available. Access to these data may be requested through the Guangdong Provincial Center for Skin Diseases and STI Control, China (contact via wangcheng090705@gmail.com) for researchers who meet the criteria for access to confidential data. We have added related information in Line 130-134.

**Results**

Reviewer #1:

The analysis presented match the analysis plan.
results clearly and completely presented
figures (Tables, Images) are of sufficient quality

Reviewer #2:

**Point1：**Lines 203-205: “A peak period”? Is there a peak monthly period each year? Or were these data aggregated?

**Response1**: Sorry for the confusion. P&S syphilis was most commonly diagnosed in June to October every year. We have rephrased this sentence to avoid misunderstanding (Line 203-204).

**Point2：**Line 207: Regarding the time series maps. It appears that the incidence rate hot spot moves from north to south regions, until the rate nearly dissolves. What is this pattern I’m seeing?

**Response2**:

Thanks for the comments. The reasons for the pattern could be, firstly, the high-risk areas were predominantly concentrated in the northern region and the south-central region before 2013. With the improvement of medical services and the implementation of screening [1], the incidence in these regions decreased. After 2017, only some coastal cities in south-central region remained a high level of morbidity. Secondly, from 2011 to 2013, new syphilis cases in eastern and western Guangdong showed a short-term increase, demonstrating a southward shift of high-risk areas in Guangdong. The temporary increase might be caused by the increase of high-risk sexual behavior after the economic development of these two regions [2-3]. At the same time, the insufficient health resources made it difficult to control the spread of syphilis caused by increased sexual transmission. For example, Maoming, the center of high incidence area in western Guangdong, had less than 0.08 health institutions per thousand population during 2011-2013, which was at the lowest level in Guangdong, implying the limited medical resources and the poor capacity to prevent the disease.

We have added the related information in the ***result*** section (Line 210-214).

References:

1. Cheng JQ, Zhou H, Hong FC, Zhang D, Zhang YJ, Pan P, et al. Syphilis screening and intervention in 500 000 pregnant women in Shenzhen, the People’s Republic of China. SEX TRANSM INFECT. 2007 2007-01-01;83(5):347-50.
2. Yang L, Tucker JD, Yang B, Shen S, Sun X, Chen Y, et al. Primary syphilis cases in Guangdong Province 1995-2008: Opportunities for linking syphilis control and regional development. BMC public health 2010;10(1):793.
3. Zhou P, Yu G, Kuang Y, Huang X, Li Y, Fu X, et al. Rapid and complicated HIV genotype expansion among high-risk groups in Guangdong Province, China. BMC INFECT DIS. 2019 2019-01-01;19(1):185.

**Point3：**Line 208: The term ‘hot spot’ is used here, but it has a specific meaning in spatial analyses that were not conducted in this study. It would be best to find a different term.

**Response3**: Thanks for the constructive comments. We have changed the word ‘hot spot’ to ‘high-risk areas’.

**Point4：**
Line 225: There is an unnecessary period after 1.17
Line 237: Change ‘autoregressive’ to ‘autoregression’
Line 254: Add ‘factors’ after sociodemographic

**Response4**: We have revised this part as the suggestion (Line 229, Line 242 and Line261).

**Point5：**Spatial clusters identification: Does the algorithm determine grouping of years, or was this done by the authors?

**Response5**: The algorithm of spatial clusters identification determines grouping of years. We have added related information in Line 167-169.

**Point6：**Results: Avoid redundancy by either 1) putting your statistics in a table OR 2) adding them to the body of the text. Generally, you should not do both. Example Line 230

**Response6**: We thank the reviewer to point out this comment. We have deleted the statistics that appear in the table from the body of the text (Line 224-253).

**Point7：**Results: I recommend that you create a map (or series of maps) that shows distribution of your variables (e.g., population density, net migration, health institutions, etc.). It would be very helpful to visualize these data spatially. It could just be an appendix or supplemental figure.

**Response7**: Thanks for the constructive comments. We have created series of maps as supplemental figures to show distribution of statistically significant variables during 2005-2017 (S2-S5 Figs).

**Point8：**Results: Can you graphically present the U-shaped relationships you found in this study?

**Response8**:

We plotted a scatter diagram of GDP per capita (RMB) in all 21 cities of Guangdong province from 2005-2017 and the incidence rates of P&S syphilis (per 100,000 population) (S1 Fig). The inverted U-shaped relationship could be graphically presented by the scatter plot.

**Editorial and Data Presentation Modifications**

Reviewer #1: Accept

Reviewer #2:

**Point1：**There is quite a bit of tense-switching throughout the manuscript (past, present, and future tenses were all noted). Please check thoroughly.
**Response1**: We have checked and revised the full text. In addition, a native English speaker helped us edit and polish the article to prevent the wrong tense and unclear presentation.

Thanks for your reconsideration.

Sincerely Yours,

Li Ling
